# Coordination difficulties, IQ and psychopathology in children with high-risk copy number variants

**DOI:** 10.1017/S0033291719003210

**Published:** 2021-01

**Authors:** Adam C. Cunningham, Jeremy Hall, Michael J. Owen, Marianne B. M. van den Bree

**Affiliations:** MRC Centre for Neuropsychiatric Genetics and Genomics, Division of Psychological Medicine and Clinical Neurosciences, Cardiff University School of Medicine, Cardiff, UK

**Keywords:** ADHD, anxiety, autism, CNV, coordination, copy number variants, development, IQ, motor skills

## Abstract

**Background:**

The prevalence and impact of motor coordination difficulties in children with copy number variants associated with neurodevelopmental disorders (ND-CNVs) remains unknown. This study aims to advance understanding of motor coordination difficulties in children with ND-CNVs and establish relationships between intelligence quotient (IQ) and psychopathology.

**Methods:**

169 children with an ND-CNV (67% male, median age = 8.88 years, range 6.02–14.81) and 72 closest-in-age unaffected siblings (controls; 55% male, median age = 10.41 years, s.d. = 3.04, range 5.89–14.75) were assessed with the Developmental Coordination Disorder Questionnaire, alongside psychiatric interviews and standardised assessments of IQ.

**Results:**

The children with ND-CNVs had poorer coordination ability (*b* = 28.98, *p* < 0.001) and 91% of children with an ND-CNV screened positive for suspected developmental coordination disorder, compared to 19% of controls (OR = 42.53, *p* < 0.001). There was no difference in coordination ability between ND-CNV genotypes (*F* = 1.47, *p* = 0.184). Poorer coordination in children with ND-CNV was associated with more attention deficit hyperactivity disorder (ADHD) (*β* = −0.18, *p* = 0.021) and autism spectrum disorder trait (*β* = −0.46, *p* < 0.001) symptoms, along with lower full-scale (*ß* = 0.21, *p* = 0.011), performance (*β* = −0.20, *p* = 0.015) and verbal IQ (*β* = 0.17, *p* = 0.036). Mediation analysis indicated that coordination ability was a full mediator of anxiety symptoms (69% mediated, *p* = 0.012), and a partial mediator of ADHD (51%, *p* = 0.001) and autism spectrum disorder trait symptoms (66%, *p* < 0.001) as well as full scale IQ (40%, *p* = 0.002), performance IQ (40%, *p* = 0.005) and verbal IQ (38%, *p* = 0.006) scores.

**Conclusions:**

The findings indicate that poor motor coordination is highly prevalent and closely linked to risk of mental health disorder and lower intellectual function in children with ND-CNVs. Future research should explore whether early interventions for poor coordination ability could ameliorate neurodevelopmental risk.

## Background

Difficulties with motor skills can have serious consequences for a child's independence and daily functioning (Van der Linde *et al*., 2015) and these negative effects can persist into adulthood (Kirby *et al*., 2013; Kirby *et al*., 2014).

Difficulties with coordinated movement are often seen in combination with other neurodevelopmental disorders such as attention deficit hyperactivity disorder (ADHD) and autism spectrum disorder (ASD). For example, it has been estimated that up to 50% of children with developmental coordination disorder (DCD), a neurodevelopmental disorder characterised by functional difficulties with coordinated movement, also possess a diagnosis of ADHD, usually of the inattentive subtype (Kadesjö and Gillberg, 1999; Kaiser *et al*., 2015). Individuals with DCD also often show problems in neurocognition, particularly in executive functioning (Wilson *et al*., 2013).

A range of genomic disorders such as those caused by sub-microscopic deletions or duplications of chromosomal regions including 1q21.1, 16p11.2 or 22q11.2 have been associated with the development of conditions such as ADHD, ASD, schizophrenia and intellectual disability (Torres *et al*., 2016; Chawner *et al*., 2019). These chromosomal abnormalities are termed copy number variants (CNVs), as they change the number of copies of genes contained on the affected area of the chromosome. While there is strong evidence that many CNVs are associated with a high risk of developing neurodevelopmental disorder (ND), including psychopathology (referred to hereafter as ND-CNVs), penetrance is often incomplete, and expressivity is variable. This means that while some individuals will display many complex symptoms, others may show few or none (Crawford *et al*., 2018).

Previous research by our group has found that ~80% of children with 22q11.2 Deletion Syndrome (22q11.2DS) show poor coordination of movement (Cunningham *et al*., 2018). Our findings also indicated that the children who showed poorer motor coordination had higher risks of ADHD, ASD and anxiety symptoms and lower mean intelligence quotient (IQ). However, there is very little research into coordination difficulties in children with other ND-CNVs. It is therefore unclear if individuals with other ND-CNVs experience similar coordination difficulties, or if certain ND-CNVs confer greater risk for coordination difficulties than others. Similarly, it is unknown if the links between coordination difficulties and other neurodevelopmental symptoms that we found for 22q11.2DS are also present in other high-risk ND-CNVs. More generally, the links between coordination difficulties and other neurodevelopmental problems are not well understood, but it has been found that CNVs found in individuals with DCD are enriched for neurodevelopmental genes (Mosca *et al*., 2016).

Finally, it is not known to what extent motor coordination disorder mediates the effects of carrying an ND-CNV on subsequent neurodevelopmental impairment. This idea is supported by theories that the early development of motor skills influences the development of other higher cognitive processes (Wilson, 2002). Motor skills develop very early in life and it follows that difficulties with interacting with and exploring one's environment will impact on the development of other skills. For example, it has been suggested that poor motor development will impair the development of skills such as the representation of abstract concepts (Piaget, 1954), mathematics (Giles *et al*., 2018) and language ability (Rowe *et al*., 2008).

With these ideas in mind, we assessed motor coordination, IQ and psychopathology in a large group of children with ND-CNVs in order to test the following hypotheses: (1) children with an ND-CNV will have poorer coordination ability (lower Developmental Coordination Disorder Questionnaire (DCDQ) scores) and will be more likely to have suspected DCD (measured by DCDQ cut-off criteria) than controls. We base this hypothesis on research where neurodevelopmental problems have been found to be associated with increased risk of motor coordination difficulties in non-genotyped samples (Kadesjö and Gillberg, 1999; Pratt and Hill, 2011; Skirbekk *et al*., 2012; Kaiser *et al*., 2015; Sumner *et al*., 2016), as well as evidence from 22q11.2DS (Cunningham *et al*., 2018); (2) motor coordination ability will differ across genotypes, as different ND-CNV's affect different genes in different areas of the genome; (3) poor coordination will be related to increased levels of psychopathology and lower IQ in children with ND-CNVs, similar to the pattern seen in non-CNV populations (Wilson *et al*., 2013; Harrowell *et al*., 2017) as well as 22q11.2DS (Cunningham *et al*., 2018) and (4) the risk of psychopathology and low IQ posed by carrying a ND-CNV is partially indirect, via motor coordination ability, or in other words, motor coordination ability will mediate the relationship between the ND-CNV status and psychopathology and IQ. This hypothesis is supported by findings that appropriate development of motor skills is required for the development of higher-order cognitive skills (Wilson, 2002; Rowe *et al*., 2008; Giles *et al*., 2018).

## Methods

### Participants

One-hundred and sixty-nine participants with a range of ND-CNVs took part (67% male, median age: 8.88 years, range: 6.02–14.81, see online Supplementary Table S1 for CNVs included), as well as 72 closest-in-age unaffected siblings (controls; 54% male, median age: 10.41 years, range: 5.89–14.75). Families were recruited through UK Medical Genetics clinics as well as word of mouth and the charities Unique and MaxAppeal!. Informed and written consent was obtained prior to recruitment from the carers of the children and recruitment was carried out in agreement with protocols approved by the London Queens Square NRES Committee. Individual ND-CNV genotypes were established from medical records as well as in-house genotyping at the Cardiff University MRC Centre for Neuropsychiatric Genetics and Genomics using microarray analysis. Information about medical comorbidities including congenital heart defects, epilepsy and premature birth, along with medication use were collected by questionnaire.

### Functional coordination impairment – the DCDQ

The DCDQ (Wilson *et al*., 2009) was completed by a single primary caregiver. It is designed to screen for functional motor coordination impairments in children 5–15 years old, and is widely used and validated (Wilson *et al*., 2009; Cunningham *et al*., 2018). In general, lower scores indicate greater coordination difficulties. Items probe fine and gross motor skills. It yields a total score as well as separate scores for three subscales: control during movement, fine motor/handwriting and general coordination. In addition, participants were categorised into those screening positive *v.* negative for ‘suspected DCD’ based on the DCDQ total score as compared with the appropriate age thresholds (Wilson *et al*., 2009). The DCDQ was used as a proxy for a more complete motor evaluation and not to primarily diagnose DCD. A diagnosis of DCD would require more in-depth motor assessments, including assessments of functional impairment.

### IQ assessment

Full scale, verbal and performance IQ (FSIQ, VIQ and PIQ) was obtained by administering the Wechsler Abbreviated Scale of Intelligence (WASI), which comprises four subtests: matrix reasoning, block design, vocabulary and similarities (Wechsler, 1999).

### Psychiatric assessment

The social communication questionnaire (SCQ) (Rutter *et al*., 2003) was used to screen for ASD trait symptoms. A score of 15 or greater is considered suggestive of ASD. The SCQ consists of three subscales: repetitive behaviour, social interactions and communication ability which are combined to give a total score.

ADHD, anxiety and ODD symptoms were assessed using the semi-structured Child and Adolescent Psychiatric Assessment (Angold *et al*., 2009). The interview was conducted by trained psychologists with the primary caregiver. Interviews were audiotaped, and DSM-5 diagnosis obtained during consensus meetings led by a child and adolescent psychiatrist. We did not consider diagnoses to be mutually exclusive. Anxiety symptoms included any symptom of generalised anxiety disorder, social phobia, specific phobia, separation anxiety, panic disorder with and without agoraphobia, agoraphobia and obsessive-compulsive disorder.

### Statistical analysis

To investigate coordination ability we conducted a linear mixed-effect model where the continuous DCDQ total score was predicted by the ND-CNV status with age as a covariate. This model included family membership as a random effect to take into account that the controls were siblings of the children with ND-CNVs.

As a sensitivity analysis, this mixed-effect model was also run with the following additional covariates: maternal education, family income, child gender, the presence of congenital heart defects, epilepsy, premature birth and the presence or absence of child medication use. This allowed us to establish whether these covariates played a role in the relationships between the DCDQ total score and ND-CNV status.

Additionally, we carried out a χ^2^ test in order to investigate the association between the presence or absence of an ND-CNV (children with an ND-CNV *v.* sibling controls) and screening positive or negative for suspected DCD.

An ANCOVA was used to investigate the extent to which the DCDQ total score was explained by the ND-CNV genotype. These analyses were based on the ND-CNV groups with 10 or more individuals available (15q11.2 deletion, 15q13.3 deletion, 16p11.2 deletion, 16p11.2 duplication, 1q21.1 deletion, 1q21.1 duplication, 22q11.2 deletion, and 22q11.2 duplication. Age was entered as a covariate. A similar ANCOVA was used to investigate the effect of deletion or duplication of the genetic material across the ND-CNV group, with age.

In order to investigate the relationship between the DCDQ score and psychopathology or IQ in children with ND-CNVs, hierarchical regression models were constructed where the DCDQ total score was predicted first by age, then the relevant psychopathology or IQ variable at the second step. For the analysis including IQ, we ran models with both standardised IQ (i.e. FSIQ, VIQ and PIQ scores) as well as raw IQ subtest scores which are not standardised by age. The latter were included because of the potential for coordination ability to impact on the IQ test performance, particularly in the block design task.

**Fig. 1.**
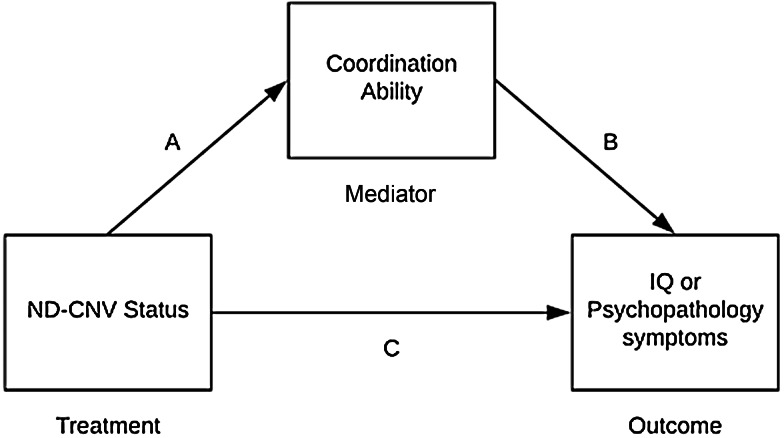
The mediation model we tested to investigate the associations between ND-CNV status (0 = sibling control; 1 = ND-CNV) and outcome, via a direct (path C) and indirect pathway (A, B). Pathways A and B estimate to what extent the link between the CNV status and outcome can be accounted for by an indirect link via a coordination ability (DCDQ Score) mediator.

Additionally, mediation analyses (Baron and Kenny, 1986) were run to investigate whether the relationship between the ND-CNV status and psychopathology or IQ scores was mediated by motor coordination ability, with age as a covariate (Fig. 1). The R package ‘mediation’ v4.4.7 (Tingley *et al*., 2014) was used to conduct the mediation analyses. This provides point estimates of the average causal mediation effects (ACME), average direct effects (ADE) and total effects (ADE + ACME) and the proportion of the total effects that are accounted for by the indirect path (proportion mediated). Confidence intervals (CIs) and *p*-values were obtained using nonparametric bootstrapping with 5000 simulations (Imai *et al*., 2010*a*). The results of the analysis can be interpreted in the following ways. If a significant total effect, and direct effect (ADE) but insignificant indirect effect (ACME) are found, the mediator has no effect on the outcome. If a significant total effect, significant indirect effect, but insignificant direct effect is found, the mediator is fully mediating the effect of the independent variable on the outcome. Finally, if a significant total effect and significant direct and indirect effects are found, the mediating variable is a partial mediator of the effect of the independent variable on the outcome. Importantly, if the total effect is not significant, there is no evidence that the independent variable has an effect on the outcome.

To validate that the mediation analyses were robust to the mediation path being investigated, a second set of models investigating if FSIQ mediated the effect of the ND-CNV status on psychopathology were also constructed and tested using the same method.

Sensitivity analyses were carried out to ensure that indirect effects were robust to violations of the assumption of sequential ignorability (effect of unmeasured confounding variables). For these sensitivity analyses, the level of confounding due to unmeasured confounders was represented by the correlation between the residuals (error terms) from the models where the mediator and outcome variables are the dependant variable, denoted *ρ* (rho). If *ρ* = 0 there is no correlation between residuals which is interpreted as no unmeasured confounding. By varying levels of *ρ* between values of −1 and +1 we can explore the likelihood that any detected indirect effect is influenced by unmeasured confounders. The key outcome of these sensitivity analyses is the value of *ρ* where the indirect effect becomes non-significant, giving a measure of how strong the effect of any unmeasured confounding variables would need to be in order to invalidate the estimated indirect effect (ACME) (Imai *et al*., 2010*b*). We used as a rule of thumb that values of *ρ* over 0.3 indicate that the detected mediation effect is robust to unmeasured confounders.

All analyses were carried out on Mac OSX and R v3.5.3. The number of individuals with information for each assessment may differ, due to not successfully completing assessments.

For all models, individuals with missing data for any variables included in the model were excluded.

## Results

Descriptive statistics for the individuals that took part in this study are presented in Table 1. Children with a ND-CNV were younger than the unaffected sibling controls but the proportion of males and females was similar. Due to this, and the fact that the DCDQ total score correlated with age (Spearman's *r* = 0.26, *p* < 0.001) in children with an ND-CNV, age was included as a covariate in all analyses. FSIQ scores could not be calculated for 12 individuals with an ND-CNV and three sibling controls, because they did not complete the WASI. A table of correlations between all quantitative variables after regressing out age from the DCDQ score (e.g. IQ and psychopathology symptom counts) is presented in online Supplementary Table S2.

**Table 1.** Demographic and summary statistics of participants

	ND-CNV (*N* = 169)	Control (*N* = 72)	*p* value
Approximate family income			
*N*-Miss	18	16	
⩽£ 19 999	59 (39.1%)	12 (21.4%)	
£ 20 000–£ 39 999	49 (32.5%)	27 (48.2%)	
£ 40 000–£ 59 999	22 (14.6%)	8 (14.3%)	
£ 60 000 +	21 (13.9%)	9 (16.1%)	
Maternal education			
*N*-Miss	5	10	
High	45 (27.4%)	16 (25.8%)	
Low	40 (24.4%)	17 (27.4%)	
Middle	70 (42.7%)	28 (45.2%)	
No school leaving exams	9 (5.5%)	1 (1.6%)	
Maternal ethnicity			
*N*-Miss	6	10	
European	150 (92.0%)	56 (90.3%)	
Other	13 (8.0%)	6 (9.7%)	
Age			<0.001^a^
*N*-Miss	0	0	
Median (Q1, Q3)	8.877 (7.116, 10.958)	10.408 (8.801, 12.364)	
Range	6.016–14.810	5.894–14.753	
Gender			0.062^b^
*N*-Miss	0	0	
Male	113 (66.9%)	39 (54.2%)	
Female	56 (33.1%)	33 (45.8%)	
Premature birth			0.010^b^
*N*-Miss	11	7	
No	72 (45.6%)	42 (64.6%)	
Yes	86 (54.4%)	23 (35.4%)	
Congenital heart defects			0.008^b^
*N*-Miss	6	2	
No	139 (85.3%)	68 (97.1%)	
Yes	24 (14.7%)	2 (2.9%)	
Epilepsy			<0.001^b^
*N*-Miss	5	2	
No	138 (84.1%)	70 (100.0%)	
Yes	26 (15.9%)	0 (0.0%)	
DCDQ total score			<0.001^a^
*N*-Miss	0	0	
Median (Q1, Q3)	32.000 (25.000, 41.000)	69.500 (58.500, 74.000)	
Range	15.000–73.000	19.000–75.000	
Control during movement score			<0.001^a^
*N*-Miss	0	0	
Median (Q1, Q3)	14.000 (10.000, 19.000)	29.000 (25.750, 30.000)	
Range	6.000–30.000	7.000–30.000	
Fine motor score			<0.001^a^
*N*-Miss	0	0	
Median (Q1, Q3)	8.000 (5.000, 11.000)	19.000 (16.500, 20.000)	
Range	4.000–20.000	4.000–20.000	
General coordination score			<0.001^a^
*N*-Miss	0	0	
Median (Q1, Q3)	10.000 (8.000, 13.000)	22.000 (17.000, 25.000)	
Range	5.000–25.000	7.000–25.000	
FSIQ			<0.001^c^
*N*-Miss	12	3	
Mean (s.d.)	81.898 (14.997)	99.246 (12.838)	
Range	51.000–128.000	70.000–139.000	
PIQ			<0.001^c^
*N*-Miss	12	3	
Mean (s.d.)	86.146 (15.276)	101.681 (13.376)	
Range	54.000–132.000	75.000–131.000	
VIQ			<0.001^c^
*N*-Miss	11	3	
Mean (s.d.)	80.835 (15.306)	97.130 (14.214)	
Range	55.000–123.000	63.000–141.000	
ADHD symptom count			<0.001^a^
*N*-Miss	0	0	
Median (Q1, Q3)	6.000 (2.000, 11.000)	0.000 (0.000, 1.000)	
Range	0.000–17.000	0.000–17.000	
ASD trait symptom count			<0.001^a^
*N*-Miss	0	0	
Median (Q1, Q3)	18.000 (11.000, 23.000)	3.000 (2.000, 6.000)	
Range	0.000–33.000	0.000–34.000	
Anxiety symptom count			<0.001^a^
*N*-Miss	0	0	
Median (Q1, Q3)	2.000 (0.000, 8.000)	0.000 (0.000, 1.000)	
Range	0.000–25.000	0.000–17.000	
ODD symptom count			<0.001^a^
*N*-Miss	0	0	
Median (Q1, Q3)	2.000 (1.000, 4.000)	0.000 (0.000, 2.000)	
Range	0.000–7.000	0.000–6.000	

a Kruskal–Wallis rank sum test.

b Pearson's χ^2^ test.

c Linear model ANOVA.

Children with an ND-CNV were taking the following medications: sodium valproate (*n* = 8), methylphenidate (*n* = 6), carbamazepine (*n* = 3), risperidone (*n* = 3), atomoxetine (*n* = 2), levetiracetam (*n* = 2), ethosuximide (*n* = 2), clobazam (*n* = 1), clobazam (*n* = 1), lamotrigine (*n* = 1), fluvoxamine (*n* = 1), guanfacine (*n* = 1), fluoxetine (*n* = 1) and nitrazepam (*n* = 1). No other relevant medication use was noted. Notably, 54% of children with an ND-CNV had a congenital heart defect, 16% a history of seizures and 54% were born before 37 weeks.

### Hypothesis 1 Do DCDQ scores differ between individuals with an ND-CNV and controls?

Children with an ND-CNV had lower DCDQ total and subscale scores than controls (Table 1). A linear mixed model where the DCDQ total score was predicted by the ND-CNV status and age with family membership as a random effect, found that the ND-CNV status was predictive of the DCDQ total score (*b* = 28.98, *p* < 0.001), along with age (*b* = 0.78, *p* = 0.022). Online Supplementary Table S3 includes mean DCDQ scores across various age and IQ ranges.

Addition of maternal education, family income, child gender, the presence of congenital heart defects, epilepsy, premature birth and child medication use in the mixed effect models as covariates indicated that they were not significant predictors of the DCDQ total score. Therefore, these were not included as covariates in any subsequent analyses.

Rates of suspected DCD were higher in individuals with an ND-CNV than controls. Ninety-one percent (154/169) of children with an ND-CNV and 19% (14/72) of controls screened positive for suspected DCD (OR = 42.53, χ^2^ = 122.86, *p* = <0.001). Across ND-CNV genotypes we studied, rates of suspected DCD were generally high. All (100%) children with 15q11.2 duplication, 16p11.2 distal duplication, 16p11.2 duplication, 1q21.1 deletion, 1q21.1 duplication, 22q11.2 distal deletion, deletion of 9q34.3 (Kleefstra Syndrome), TAR deletion and TAR duplication screened positive (online Supplementary Table S1).

### Hypothesis 2 Does coordination score differ by genotype?

After including age, the ND-CNV genotype was not a significant predictor of the DCDQ score (*F* = 1.47, df = 7, *p* = 0.184, *η*^2^ = 0.069) in those ND-CNV groups with 10 individuals or more. These findings are consistent with the null hypothesis that coordination ability is similar regardless of the ND-CNV genotype. In addition, type of CNV (i.e. deletion (*n* = 101) or duplication (*n* = 68) of the chromosomal material) had no effect on the DCDQ score (*F* = 0.67, df = 1, *p* = 0.413, *η*^2^ = 0.003).

### Hypothesis 3 Is coordination related to psychopathology and IQ in children with an ND-CNV?

Children with ND-CNV's displayed higher levels of psychopathology symptoms than siblings (Table 1). When investigating the links between coordination difficulties and psychopathology within the ND-CNV group, we found that worse coordination ability was associated with a greater number of ADHD and ASD trait symptoms, but not anxiety or ODD symptoms. In all models, age was a significant covariate, with older children having better coordination ability (Table 2).

**Table 2.** Regression results for the DCDQ score predicted by (A) ADHD symptom counts, (B) ASD trait symptom count, (C) anxiety symptoms and (D) oppositional defiant disorder (ODD) symptoms, with age as a covariate

Predictors	DCDQ Score		
	*β*	95% CI	*p*
(A)			
Age	0.20	0.05–0.34	**0.009**
ADHD symptom count	−0.18	−0.32 to −0.03	**0.021**
Observations		169	
*R*^2^/adjusted *R*^2^		0.078/0.067	
(B)			
Age	0.16	0.02–0.29	**0.021**
ASD traits symptom count	−0.46	−0.59 to −0.32	**<0.001**
Observations		169	
*R*^2^/adjusted *R*^2^		0.252/0.243	
(C)			
Age	0.22	0.07–0.37	**0.004**
Anxiety symptom count	−0.11	−0.26 to 0.04	0.152
Observations		169	
*R*^2^/adjusted *R*^2^		0.059/0.048	
(D)			
Age	0.22	0.07–0.37	**0.004**
ODD symptom count	0.02	−0.13 to 0.17	0.767
Observations		169	
*R*^2^/adjusted *R*^2^		0.048/0.036	

Bolded values indicate significant at *p* < 0.05.

Average FSIQ, PIQ and VIQ were lower in the ND-CNV group (Table 1). Within children with ND-CNV's, worse coordination was associated with lower FSIQ (*β* = 0.21, *p* = 0.011), PIQ (*β* = 0.20, *p* = 0.015) and VIQ (*β* = 0.036, *p* = 0.036), with age as a significant covariate (online Supplementary Table S4A–C). When investigating if the DCDQ score was associated with raw subtest scores, we found that poorer matrix reasoning (*β* = 0.18, *p* = 0.041), but not block design, similarities or vocabulary performance was associated with poorer coordination (online Supplementary Table S4D–G). In the ND-CNV group, there was no difference in DCDQ total scores between individuals with missing FSIQ scores and individuals with complete FSIQ data (*W* = 715, *p* = 0.165).

### Hypothesis 4 Do coordination difficulties mediate the relationship between ND-CNV group status and psychopathology or IQ?

Mediation analysis indicated that coordination ability (DCDQ total score) was a full mediator of the effect of having an ND-CNV on anxiety symptoms (69% mediated) and a partial mediator of both ADHD symptoms (51% mediated) and ASD trait symptoms (66% mediated). No evidence for mediation was found for ODD symptoms (Table 3).

**Table 3.** Results of mediation analysis on the effect of having an ND-CNV on (A) ADHD symptom counts, (B) ASD trait symptom count, (C) anxiety symptoms and (D) ODD symptoms with coordination ability as a mediator

	Estimates	Lower 95% CI	Upper 95% CI	*p*
(A) ADHD symptom counts				
Average causal mediation effects	2.54	1.04	4.24	0.001
Average direct effects	2.39	0.13	4.44	0.042
Total effects	4.93	3.67	6.10	<0.001
Prop. mediated	0.51	0.20	0.97	0.001
(B) ASD trait symptom counts				
Average causal mediation effects	8.13	6.14	10.44	<0.001
Average direct effects	4.14	1.16	6.95	0.005
Total effects	12.27	10.44	14.06	<0.001
Prop. mediated	0.66	0.48	0.90	<0.001
(C) Anxiety symptom counts				
Average causal mediation effects	1.84	0.35	3.45	0.012
Average direct effects	0.81	−1.24	2.79	0.428
Total effects	2.65	1.42	3.89	<0.001
Prop. mediated	0.69	0.12	1.71	0.012
(D) ODD symptom counts				
Average causal mediation effects	0.27	−0.26	0.80	0.304
Average direct effects	0.91	0.16	1.66	0.017
Total effects	1.18	0.70	1.64	<0.001
Prop. mediated	0.23	−0.22	0.82	0.304

Average causal mediation effects corresponds to the indirect effect through coordination ability, while average direct effects correspond to the direct effect of having an ND-CNV on the psychopathology variable of interest.

Sensitivity analysis indicated that the detected mediation effect of coordination ability on ASD traits was robust to unmeasured confounding variables (*ρ* > 0.49). However, we found lower values for *ρ* in mediation models for ADHD (*ρ* > 0.24) and anxiety symptoms (*ρ* > 0.16), suggesting the mediation effects in these models may be more sensitive to confounding.

Mediation analysis also revealed that coordination ability was a partial mediator of FSIQ, PIQ and VIQ scores, mediating 40% of the effect of having an ND-CNV on FSIQ and PIQ, and 38% of the effect on VIQ (Table 4). However, we found low values of *ρ* for these models, *ρ* > 0.21 for FSIQ, *ρ* > 0.17 for VIQ and *ρ* > 0.19 for PIQ, suggesting that these models may be sensitive to confounding.

**Table 4.** Results of mediation analysis on the effect of having an ND-CNV on (A) full scale IQ, (B) performance IQ and (C) verbal IQ with coordination ability as a mediator

	Estimates	Lower 95% CI	Upper 95% CI	*p*
(A) FSIQ				
Average causal mediation effects	−7.12	−12.17	−2.58	0.002
Average direct effects	−10.86	−17.06	−4.81	<0.001
Total effects	−17.98	−22.02	−13.98	<0.001
Prop. mediated	0.40	0.14	0.70	0.002
(B) PIQ				
Average causal mediation effects	−6.68	−11.79	−2.08	0.005
Average direct effects	−9.89	−16.03	−3.39	0.005
Total effects	−16.57	−20.76	−12.39	<0.001
Prop. mediated	0.40	0.12	0.76	0.005
(C) VIQ				
Average causal mediation effects	−6.21	−11.14	−1.81	0.006
Average direct effects	−10.28	−16.66	−3.75	0.004
Total effects	−16.49	−20.71	−12.18	<0.001
Prop. mediated	0.38	0.11	0.74	0.006

Average causal mediation effects correspond to the indirect effect through coordination ability, while average direct effects correspond to the direct effect of having an ND-CNV on the psychopathology variable of interest.

Previous studies have found no evidence that IQ is associated with levels of psychopathology in children with an ND-CNV (Niarchou *et al*., 2014). In order to validate that the mediation analyses were robust, we constructed a second set of models where FSIQ was included instead of coordination ability as a mediator of the effect of the ND-CNV status on psychopathology. In agreement with the previous findings, we found no evidence that FSIQ was a mediator of ADHD (ACME = 0.25, *p* = 0.549), ASD traits (ACME = 0.44, *p* = 0.466), anxiety (ACME = 0.22, *p* = 0.574) or ODD symptoms (ACME = 0.44, *p* = 0.478).

## Discussion

This study shows that difficulties with coordination are common in children with ND-CNVs, with individuals with an ND-CNV having lower DCDQ total scores than controls, and a large proportion (91%) of children with an ND-CNV screening positive for suspected DCD. We also present evidence that coordination ability is associated with increased ADHD and ASD traits, and lower FSIQ, PIQ and VIQ scores. Coordination difficulties were elevated across all ND-CNV genotypes and genotype or CNV type (deletion or duplication) was not a significant predictor of coordination ability. Importantly, we find that coordination ability (DCDQ total score) is a partial mediator of the effect of ADHD symptoms and ASD traits, along with FSIQ, PIQ and VIQ, and a full mediator of anxiety symptoms, while we found no evidence for mediation of ODD symptoms.

The high rates of coordination difficulties across genotypes, and the lack of specificity of ND-CNV genotype on coordination ability, indicates that neuromotor deficits are a common outcome across ND-CNVs. We found no evidence for an effect of gender on coordination ability, which differs from studies in the general population where DCD is more commonly seen in boys (Tsiotra *et al*., 2006; Missiuna *et al*., 2008). It is important to note that rates of premature birth were also high in the ND-CNV group at 54%. Prematurity has been linked to delays in motor development and coordination difficulties later in life (Goyen and Lui, 2009; Edwards *et al*., 2011), but we found no effect of prematurity on the total DCD score. Additionally, while 19% of controls screened positive for suspected DCD, this is within the range of prevalence estimates for developmental coordination disorder in the general population, which range from 2–20%, depending on criteria used (Blank *et al*., 2019).

Considering links with psychopathology, children with a ND-CNV showed elevated ADHD, ASD trait, anxiety and ODD symptoms compared to controls. Within the ND-CNV group, higher numbers of ADHD and ASD symptoms were associated with greater motor coordination difficulties (lower DCDQ total score), but this was not the case for anxiety or ODD. These results are similar to research in non-genotype selective samples, where high rates of coordination difficulties have been observed in children with ADHD (Watemberg *et al*., 2007) and/or ASD (Sumner *et al*., 2016), but differ from findings in children with 22q11.2 deletion, where an association with anxiety was found (Cunningham *et al*., 2018).

Additionally, the total DCDQ score was found to be a partial mediator of the effect of the ND-CNV status on ADHD symptoms and ASD traits and a full mediator of anxiety symptoms. No evidence for mediation was found for ODD symptoms. These results may suggest that coordination ability is intrinsically linked to the development of ADHD, ASD traits and anxiety symptoms, but not ODD symptoms. However, it is important to note that the identified mediating effects on ADHD and anxiety symptoms may need to be viewed with caution, as the models had reduced robustness against unmeasured confounding.

Importantly, there was no evidence that FSIQ mediated the effect of an ND-CNV on psychopathology. This agrees with previous work in 22q11.2 deletion, where it was found that FSIQ was not associated with levels of psychopathology (Niarchou *et al*., 2014). This helps us validate that our mediation analysis is not prone to detecting false positive mediating effects.

Poor coordination was also found to be associated with FSIQ, VIQ and PIQ in the ND-CNV group. This agrees with previous studies of children with coordination difficulties, not selected due to genotype, and with previous research in 22q11.2DS (Roizen *et al*., 2011; Cunningham *et al*., 2018), where associations between FSIQ and motor performance have been found. The presented results, and previous research in 22q11.2DS, support the idea that within ID populations, the level of intellectual impairment is associated with poorer motor coordination (Vuijk *et al*., 2010). It is of note that we found significant associations between coordination ability and raw scores on the matrix reasoning task, while no relationships were found between coordination ability and the block design, vocabulary or similarities tasks. The lack of association between DCDQ total scores and block design performance is somewhat unexpected as this WASI subtest requires movement (e.g. placing blocks in the correct place to complete a pattern). Our finding may suggest that in this population, performance on this task depends more on spatial reasoning than ability to manipulate the blocks.

While associations between vocabulary and gesture performance (a form of coordinated movement) have been found in infants, these relationships have not been thoroughly investigated in older children. We did not find any association between the vocabulary subtest performance and DCDQ total score, and it may be that the relationships between motor skills and verbal ability are weaker in older children. In addition, the vocabulary task does not have a movement component, so is unlikely to be confounded by poor coordination ability.

Mediation analysis also found that coordination ability was a partial mediator of the effect of the ND-CNV status on FSIQ, VIQ and PIQ. This agrees with theories that suggest that motor skills are required for the development of higher order cognitive skills, such as mathematical ability (Giles *et al*., 2018), which would be accounted for in PIQ or language development (Rowe *et al*., 2008) as accounted for by VIQ.

A number of different interpretations of our mediation findings are possible. First, aberrant development of motor coordination skills as a consequence of a ND-CNV may have cascading impacts on later development of other skills, like cognition, attention and social functioning. Second, motor coordination difficulties trigger environmental risk factors, such as social exclusion and bullying, which may increase the risk of psychopathology. Thirdly, both motor coordination problems and impairment in IQ and psychopathology may be the result of the same underlying genetic cause (pleiotropy), although the manifestation of the symptoms may occur at different stages during development. Such pleiotropic effects probably impact on brain development. For example, the cerebellum has been shown to be important in many motor as well as non-motor functions (Miall *et al*., 2001; Stoodley, 2016) and, therefore, damage to this region has cross domain effects. It is also possible that multiple processes account for the findings, possibly at different developmental stages and in different ways for each trait. It is for example noteworthy that we did not find evidence of mediation of motor coordination problems for ODD.

We are not able to further distinguish between these hypotheses in the current study, and the cross-sectional design does not allow for insights into order of appearance of different impairments, but delays in the development of motor skills are often observed from very early in development. It would be fruitful to conduct a randomised control trial where an intervention for motor coordination difficulties is delivered at an early age to a high-risk group of children with ND-CNVs and changes in psychopathology and cognitive function are established.

This is the only study to investigate the relationships between coordination difficulties and psychopathology and IQ in children with CNV's that confer risk for psychiatric disorders. The presence of sibling controls for comparison, and detailed assessment of psychopathology are additional strengths. However, the DCDQ is a measure of overall coordination that is completed by a primary carer, and it therefore cannot allow for direct insights to be gained into underlying sensorimotor deficits that are present in the individual children. In addition, we are unable to distinguish sufficiently between motor difficulties due to a global motor delay, or due to a specific DCD like syndrome. To address these limitations, further research should investigate quality of movement using kinematic and clinical assessments of movement. An additional limitation is that the control group was significantly older than the ND-CNV group. However, this should have had limited effect on screening positive or negative for suspected DCD, as parents are always asked to rate children with respect to their peers, and screening is based on age dependent thresholds. Younger children require lower scores to screen positive than older children, allowing for a degree of internal control for improvements in ability due to age. In addition, age was included as a covariate in all analyses. It was not possible to conduct full physical and neurological assessments on the children. We did include congenital heart defects and epilepsy as covariates in our analyses, but we cannot rule out that other physical or neurological problems may affect coordination.

In summary, we found high rates of coordination difficulties in children with neurodevelopmental risk CNVs which constitute a significant portion of the caseload of clinical geneticists. These problems were elevated across all ND-CNV genotypes and associated with risk of IQ impairment and psychopathology. Furthermore, the association between ND-CNVs and anxiety symptoms was fully mediated by coordination ability, while the association between having an ND-CNV and lower FSIQ, PIQ, and VIQ scores, along with ADHD symptoms and ASD traits was partially mediated by coordination ability. The immediate clinical implication of these findings should be increased vigilance for motor impairments in children with ND-CNVs, so that appropriate support can be introduced as early as possible. It may be that motor interventions could help the development of cognitive skills and reduce risk for the development of psychopathology.

## Supplementary material

For supplementary material accompanying this paper visit https://doi.org/10.1017/S0033291719003210. click here to view supplementary material

## References

[ref1] Angold A, Prendergast M, Cox A, Harrington R, Simonoff E and Rutter M (2009) The Child and Adolescent Psychiatric Assessment (CAPA). Psychological Medicine 25, 739 10.1017/S003329170003498X. 7480451

[ref2] Baron RM and Kenny DA (1986) The moderator–mediator variable distinction in social psychological research: conceptual, strategic, and statistical considerations. Journal of Personality and Social Psychology 51, 1173–1182. 10.1037/0022-3514.51.6.1173. 3806354

[ref3] Blank R, Barnett AL, Cairney J, Green D, Kirby A, Polatajko H, Rosenblum S, Smits‐Engelsman B, Sugden D, Wilson P and Vinçon S (2019) International clinical practice recommendations on the definition, diagnosis, assessment, intervention, and psychosocial aspects of developmental coordination disorder. Developmental Medicine & Child Neurology 61, 242–285. 10.1111/dmcn.14132. 30671947 PMC6850610

[ref4] Chawner SJRA, Owen MJ, Holmans P, Raymond FL, Skuse D, Hall J and van den Bree MBM (2019) Genotype–phenotype associations in children with copy number variants associated with high neuropsychiatric risk in the UK (IMAGINE-ID): a case-control cohort study. Lancet Psychiatry 6, 493–505. 10.1016/S2215-0366(19)30123-3. 31056457

[ref5] Crawford K, Bracher-Smith M, Owen D, Kendall KM, Rees E, Pardiñas AF, Einon M, Escott-Price V, Walters JTR, O'Donovan MC, Owen MJ and Kirov G (2019) Medical consequences of pathogenic CNVs in adults: analysis of the UK Biobank. Journal of Medical Genetics 56, 131–138. 10.1136/jmedgenet-2018-105477. 30343275

[ref6] Cunningham AC, Delport S, Cumines W, Busse M, Linden DEJ, Hall J, Owen MJ and van den Bree MBM (2018) Developmental coordination disorder, psychopathology and IQ in 22q11.2 deletion syndrome. The British Journal of Psychiatry 212, 27–33. 10.1192/bjp.2017.6. 29433607 PMC6457162

[ref7] Edwards J, Berube M, Erlandson K, Haug S, Johnstone H, Meagher M, Sarkodee-Adoo S and Zwicker JG (2011) Developmental coordination disorder in school-aged children born very preterm and/or at very low birth weight: a systematic review. Journal of Developmental and Behavioral Pediatrics 32, 678–687. 10.1097/DBP.0b013e31822a396a. 21900828

[ref8] Giles OT, Shire KA, Hill LJB, Mushtaq F, Waterman A, Holt RJ, Culmer PR, Williams JHG, Wilkie RM and Mon-Williams M (2018) Hitting the target: mathematical attainment in children is related to interceptive-timing ability. Psychological Science 29, 1334–1345. 10.1177/0956797618772502. 29990446 PMC6088501

[ref9] Goyen TA and Lui K (2009) Developmental coordination disorder in ‘apparently normal’ schoolchildren born extremely preterm. Archives of Disease in Childhood 94, 298–302. 10.1136/adc.2007.134692. 18838419

[ref10] Harrowell I, Hollén L, Lingam R and Emond A (2017) Mental health outcomes of developmental coordination disorder in late adolescence. Developmental Medicine & Child Neurology 59, 973–979. 10.1111/dmcn.13469. 28512766 PMC5573907

[ref11] Imai K, Keele L and Tingley D (2010 *a*) A general approach to causal mediation analysis. Psychological Methods 15, 309–334. 10.1037/a0020761. 20954780

[ref12] Imai K, Keele L and Yamamoto T (2010 *b*) Identification, inference and sensitivity analysis for causal mediation effects. Statistical Science 25, 51–71. 10.1214/10-STS321.

[ref13] Kadesjö B and Gillberg C (1999) Developmental coordination disorder in Swedish 7-year-old children. Journal of the American Academy of Child and Adolescent Psychiatry 38, 820–828. 10.1097/00004583-199907000-00011. 10405499

[ref14] Kaiser M-LM-LL, Schoemaker MMM, Albaret J-MJ-MM and Geuze RHH (2015) What is the evidence of impaired motor skills and motor control among children with attention deficit hyperactivity disorder (ADHD)? Systematic review of the literature. Research in Developmental Disabilities 36, 338–357. 10.1016/j.ridd.2014.09.023. 25462494

[ref15] Kirby A, Williams N, Thomas M and Hill EL (2013) Self-reported mood, general health, wellbeing and employment status in adults with suspected DCD. Research in Developmental Disabilities 34, 1357–1364. 10.1016/j.ridd.2013.01.003. 23417140

[ref16] Kirby A, Sugden D and Purcell C (2014) Diagnosing developmental coordination disorders. Archives of Disease in Childhood 99, 292–296. 10.1136/archdischild-2012-303569. 24255567

[ref17] Miall RC, Reckess GZ and Imamizu H (2001) The cerebellum coordinates eye and hand tracking movements. Nature Neuroscience 4, 638–644. 10.1038/88465. 11369946

[ref18] Missiuna C, Gaines R, McLean J, Delaat D, Egan M and Soucie H (2008) Description of children identified by physicians as having developmental coordination disorder. Developmental Medicine and Child Neurology 50, 839–844. 10.1111/j.1469-8749.2008.03140.x. 18811713

[ref19] Mosca SJ, Langevin LM, Dewey D, Innes AM, Lionel AC, Marshall CC, Scherer SW, Parboosingh JS and Bernier FP (2016) Copy-number variations are enriched for neurodevelopmental genes in children with developmental coordination disorder. Journal of Medical Genetics 53, 812–819. 10.1136/jmedgenet-2016-103818. 27489308

[ref20] Niarchou M, Zammit S, van Goozen SH, Thapar A, Tierling HM, Owen MJ and van den Bree MB (2014) Psychopathology and cognition in children with 22q11.2 deletion syndrome. British Journal of Psychiatry 204, 46–54. 10.1192/bjp.bp.113.132324. PMC3877833 24115343

[ref21] Piaget J (1954) The Construction of Reality in the Child. New York: Basic Books 10.1037/11168-000.

[ref22] Pratt ML and Hill EL (2011) Anxiety profiles in children with and without developmental coordination disorder. Research in Developmental Disabilities 32, 1253–1259. 10.1016/j.ridd.2011.02.006. 21377831

[ref23] Roizen NJ, Higgins AM, Antshel KM, Fremont W, Shprintzen R and Kates WR (2011) 22q11.2 deletion syndrome: are motor deficits more than expected for IQ level? The Journal of Pediatrics 157, 658–661. 10.1016/j.jpeds.2010.04.073.22q11.2. PMC2936811 20646714

[ref24] Rowe ML, Özçalışkan Ş and Goldin-Meadow S (2008) Learning words by hand: gesture's role in predicting vocabulary development. First Language 28, 182–199. 10.1177/0142723707088310. 19763249 PMC2745165

[ref25] Rutter M, Bailey A and Lord C (2003) The Social Communication Questionnaire manual. Los Angeles: Western Psychological Services.

[ref26] Skirbekk B, Hansen BH, Oerbeck B, Wentzel-Larsen T and Kristensen H (2012) Motor impairment in children with anxiety disorders. Psychiatry Research 198, 135–139. 10.1016/j.psychres.2011.12.008. 22386219

[ref27] Stoodley CJ (2016) The cerebellum and neurodevelopmental disorders. Cerebellum 15, 34–37. 10.1007/s12311-015-0715-3. 26298473 PMC4811332

[ref28] Sumner E, Leonard HC and Hill EL (2016) Overlapping phenotypes in autism spectrum disorder and developmental coordination disorder: a cross-syndrome comparison of motor and social skills. Journal of Autism and Developmental Disorders 46, 2609–2620. 10.1007/s10803-016-2794-5. 27126816 PMC4938850

[ref29] Tingley D, Yamamoto T, Hirose K, Keele L and Imai K (2014) Mediation: R package for causal mediation analysis. Journal of Statistical Software 59, 1–38.26917999

[ref30] Torres F, Barbosa M and Maciel P (2016) Recurrent copy number variations as risk factors for neurodevelopmental disorders: critical overview and analysis of clinical implications. Journal of Medical Genetics 53, 73–90. 10.1136/jmedgenet-2015-103366. 26502893

[ref31] Tsiotra GD, Flouris AD, Koutedakis Y, Faught BE, Nevill AM, Lane AM and Skenteris N (2006) A comparison of developmental coordination disorder prevalence rates in Canadian and Greek children. Journal of Adolescent Health 39, 125–127. 10.1016/j.jadohealth.2005.07.011. 16781974

[ref32] Van der Linde BW, van Netten JJ, Otten B, Postema K, Geuze RH and Schoemaker MM (2015) Activities of daily living in children with developmental coordination disorder: performance, learning, and participation. Physical Therapy 95, 1496–1506. 10.2522/ptj.20140211. 26045605

[ref33] Vuijk PJ, Hartman E, Scherder E and Visscher C (2010) Motor performance of children with mild intellectual disability and borderline intellectual functioning. Journal of Intellectual Disability Research 54, 955–965. 10.1111/j.1365-2788.2010.01318.x. 20854287

[ref34] Watemberg N, Waiserberg N, Zuk L and Lerman-Sagie T (2007) Developmental coordination disorder in children with attention-deficit-hyperactivity disorder and physical therapy intervention. Developmental Medicine and Child Neurology 49, 920–925. 10.1111/j.1469-8749.2007.00920.x. 18039239

[ref35] Wechsler D (1999) WASI: Wechsler Abbreviated Scale of Intelligence. San Antonio, TX: Psychological Corporation.

[ref36] Wilson M (2002) Six views of embodied cognition. Psychonomic Bulletin & Review 9, 625–636. 10.3758/BF03196322. 12613670

[ref37] Wilson BN, Crawford SG, Green D, Roberts G, Aylott A and Kaplan BJ(2009) Psychometric properties of the revised Developmental Coordination Disorder Questionnaire. Physical & Occupational Therapy in Pediatrics 29, 182–202. 10.1080/01942630902784761. 19401931

[ref38] Wilson PH, Ruddock S, Smits-Engelsman B, Polatajko H and Blank R (2013) Understanding performance deficits in developmental coordination disorder: a meta-analysis of recent research. Developmental Medicine and Child Neurology 55, 217–228. 10.1111/j.1469-8749.2012.04436.x. 23106668

