# Supplementary material for: Coordination difficulties, IQ and psychopathology in children with high-risk copy number variants
Source: Psychol Med. 2019 Nov 19;51(2):290–9. doi: 10.1017/S0033291719003210 (PMC7234895; doi:10.1017/S0033291719003210)
Supplement: Supplementary file 1 [file S0033291719003210sup.zip › S0033291719003210sup001.docx]

Supplementary Table 3. Mean total and subscale scores on the DCDQ across different A) age and B) IQ ranges in individuals with an ND-CNV.

| **A)** |  |  |  |  |  |  |
| --- | --- | --- | --- | --- | --- | --- |
| **DCDQ Total Score** | **6-7.9 (N=67)** | **8-9.9 (N=41)** | **10-11.9 (N=33)** | **12-13.9 (N=22)** | **14-15.9 (N=6)** | **Total (N=169)** |
| **Mean (SD)** | 31.02 (11.85) | 36.29 (13.26) | 38.24 (13.73) | 38.27 (12.49) | 31.83 (15.36) | 34.68 (13.04) |
| **Range** | 15 - 68 | 16 - 73 | 19 - 66 | 16 - 72 | 15 – 55 | 15 - 73 |
| **Control during movement** |  |  |  |  |  |  |
| **Mean (SD)** | 13.34 (5.62) | 15.56 (6.81) | 16.64 (6.46) | 15.68 (5.96) | 15.17 (7.96) | 14.89 (6.28) |
| **Range** | 6 - 30 | 6 - 30 | 6 - 30 | 6 - 29 | 6 - 26 | 6 - 30 |
| **Fine motor** |  |  |  |  |  |  |
| **Mean (SD)** | 7.64 (3.71) | 9.17 (3.73) | 9.88 (5.09) | 10.64 (4.80) | 6.50 (3.21) | 8.80 (4.27) |
| **Range** | 4 - 18 | 4 - 19 | 4 - 20 | 5 - 20 | 4 - 11 | 4 - 20 |
| **General coordination** |  |  |  |  |  |  |
| **Mean (SD)** | 10.03 (3.89) | 11.56 (4.82) | 11.73 (4.44) | 11.96 (4.59) | 10.17 (5.19) | 10.99 (4.40) |
| **Range** | 5 - 25 | 5 - 25 | 5 - 22 | 5 - 24 | 5 - 19 | 5 - 25 |
| **B)** |  |  |  |  |  |  |
| **DCDQ Total Score** | **Moderate Intellectual Disability** **(IQ<55) (N=3)** | **Mild Intellectual Disability (56-70) (N=28)** | **Borderline Intellectual Disability (71-85) (N=78)** | **Average IQ (85-115) (N=44)** | **Above Average IQ (>115) (N=4)** | **Total (N=157)** |
| **Mean (SD)** | 35.33 (15.31) | 36.07 (12.24) | 33.53 (12.15) | 35.66 (13.63) | 44.00 (11.34) | 34.88 (12.60) |
| **Range** | 18 - 47 | 16 - 73 | 15 - 72 | 16 - 65 | 38 - 61 | 15 - 73 |
| **Control during movement** |  |  |  |  |  |  |
| **Mean (SD)** | 17 (9.64) | 15.68 (6.25) | 14.12 (5.70) | 15.66 (6.96) | 18.75 (4.99) | 15 (6.24) |
| **Range** | 6 - 24 | 6 - 30 | 6 - 29 | 6 - 30 | 15 - 26 | 6 - 30 |
| **Fine motor** |  |  |  |  |  |  |
| **Mean (SD)** | 8.67 (5.69) | 9.11 (4.37) | 8.63 (4.24) | 8.60 (3.96) | 13.50 (3.00) | 8.83 (4.21) |
| **Range** | 4 - 15 | 5 - 20 | 4 - 20 | 4 - 20 | 12 - 18 | 4 - 20 |
| **General coordination** |  |  |  |  |  |  |
| **Mean (SD)** | 9.67 (1.53) | 11.29 (4.25) | 10.78 (4.15) | 11.41 (4.57) | 11.75 (3.86) | 11.05 (4.22) |
| **Range** | 8 - 11 | 5 - 24 | 5 - 25 | 5 - 25 | 8 - 17 | 5 - 25 |
