# Supplementary material for: Coordination difficulties, IQ and psychopathology in children with high-risk copy number variants
Source: Psychol Med. 2019 Nov 19;51(2):290–9. doi: 10.1017/S0033291719003210 (PMC7234895; doi:10.1017/S0033291719003210)
Supplement: Supplementary file 1 [file S0033291719003210sup.zip › S0033291719003210sup002.docx]

Supplementary Table 4. Regression results for DCDQ score predicted by A) Full Scale IQ (FSIQ), B) Performance IQ (PIQ), C) Verbal IQ, D) Block design raw score, E) Matrix reasoning raw score, F) Similarities raw score G) Vocabulary raw score, with age as a covariate.

| **A)** | | **DCDQ Score** | |
| --- | --- | --- | --- |
| *Predictors* | *ß* | *95% CI* | *p* |
| Age | .23 | .08 – .39 | **.004** |
| FSIQ | .21 | .05 – .36 | **.011** |
| Observations | | 157 | |
| R^2^ / adjusted R^2^ | | .074 / .062 | |

| **B)** | | **DCDQ Score** | |
| --- | --- | --- | --- |
| *Predictors* | *ß* | *95% CI* | *p* |
| Age | .24 | .08 – .40 | **.004** |
| PIQ | .20 | .04 – .36 | **.015** |
| Observations | | 157 | |
| R^2^ / adjusted R^2^ | | .070 / .058 | |

| **C)** | | **DCDQ Score** | |
| --- | --- | --- | --- |
| *Predictors* | *ß* | *95% CI* | *p* |
| Age | .21 | .06 – .37 | **.007** |
| VIQ | .17 | .01 – .32 | **.036** |
| Observations | | 158 | |
| R^2^ / adjusted R^2^ | | .063 / .051 | |

| **D)** | | **DCDQ Score** | |
| --- | --- | --- | --- |
| *Predictors* | *ß* | *95% CI* | *p* |
| Age | .14 | -.03 – .31 | .101 |
| Block design | .15 | -.02 – .32 | .077 |
| Observations | | 155 | |
| R^2^ / adjusted R^2^ | | .058 / .046 | |

| **E)** | | **DCDQ Score** | |
| --- | --- | --- | --- |
| *Predictors* | *ß* | *95% CI* | *p* |
| Age | .12 | -.05 – .29 | .161 |
| Matrix reasoning | .18 | .01 – .35 | **.041** |
| Observations | | 155 | |
| R^2^ / adjusted R^2^ | | .065 / .052 | |

| **F)** | | **DCDQ Score** | |
| --- | --- | --- | --- |
| *Predictors* | *ß* | *95% CI* | *p* |
| Age | .12 | -.05 – .29 | .177 |
| Similarities | .17 | -.00 – .35 | .053 |
| Observations | | 156 | |
| R^2^ / adjusted R^2^ | | .064 / .052 | |

| **G)** | | **DCDQ Score** | |
| --- | --- | --- | --- |
| *Predictors* | *ß* | *95% CI* | *p* |
| Age | .15 | -.02 – .32 | .087 |
| Vocabulary | .13 | -.04 – .30 | .124 |
| Observations | | 156 | |
| R^2^ / adjusted R^2^ | | .056 / .043 | |
