# Supplementary material for: Coordination difficulties, IQ and psychopathology in children with high-risk copy number variants
Source: Psychol Med. 2019 Nov 19;51(2):290–9. doi: 10.1017/S0033291719003210 (PMC7234895; doi:10.1017/S0033291719003210)
Supplement: Supplementary file 1 [file S0033291719003210sup.zip › S0033291719003210sup004.docx]

Supplementary Table 2. Correlations between developmental coordination disorder questionnaire total score and other quantitative variables for A) individuals with an ND-CNV, B) controls and C) the total sample, after age has been regressed out of DCDQ total score.

| *A)* | DCDQ total score | FSIQ | VIQ | PIQ | ADHD symptom Count | ASD trait symptom count | Anxiety symptom count | ODD symptom count |
| --- | --- | --- | --- | --- | --- | --- | --- | --- |
| DCDQ total score |  | 0.208^**^ | 0.172^*^ | 0.198^*^ | -0.177^*^ | -0.460^***^ | -0.111 | 0.023 |
| FSIQ |  |  | 0.909^***^ | 0.902^***^ | 0.023 | -0.027 | -0.007 | 0.056 |
| VIQ |  |  |  | 0.649^***^ | -0.007 | -0.104 | 0.008 | 0.024 |
| PIQ |  |  |  |  | 0.049 | 0.039 | -0.019 | 0.086 |
| ADHD symptom Count |  |  |  |  |  | 0.393^***^ | 0.389^***^ | 0.413^***^ |
| ASD Trait symptom count |  |  |  |  |  |  | 0.347^***^ | 0.158^*^ |
| Anxiety symptom count |  |  |  |  |  |  |  | 0.334^***^ |
| ODD symptom count |  |  |  |  |  |  |  |  |
| Computed correlation used Pearson-method with pairwise-deletion, *p<.05, **p<.01, ***p<.001. DCDQ: Developmental Coordination Disorder Questionnaire, FSIQ: Full Scale IQ, VIQ: Verbal IQ, PIQ: Performance IQ, ADHD: Attention Deficit Hyperactivity Disorder, ASD: Autism Spectrum Disorder, ODD: Oppositional Defiant Disorder*.* | | | | | | | | |

| *B)* | DCDQ total score | FSIQ | VIQ | PIQ | ADHD symptom Count | ASD trait symptom count | Anxiety symptom count | ODD symptom count |
| --- | --- | --- | --- | --- | --- | --- | --- | --- |
| DCDQ total score |  | 0.319^**^ | 0.260^*^ | 0.276^*^ | -0.455^***^ | -0.562^***^ | -0.332^**^ | -0.340^**^ |
| FSIQ |  |  | 0.871^***^ | 0.811^***^ | -0.256^*^ | -0.115 | -0.152 | -0.044 |
| VIQ |  |  |  | 0.427^***^ | -0.278^*^ | -0.152 | -0.196 | -0.091 |
| PIQ |  |  |  |  | -0.136 | -0.015 | -0.038 | 0.049 |
| ADHD symptom Count |  |  |  |  |  | 0.656^***^ | 0.606^***^ | 0.586^***^ |
| ASD Trait symptom count |  |  |  |  |  |  | 0.524^***^ | 0.541^***^ |
| Anxiety symptom count |  |  |  |  |  |  |  | 0.560^***^ |
| ODD symptom count |  |  |  |  |  |  |  |  |
| Computed correlation used Pearson-method with pairwise-deletion, *p<.05, **p<.01, ***p<.001. DCDQ: Developmental Coordination Disorder Questionnaire, FSIQ: Full Scale IQ, VIQ: Verbal IQ, PIQ: Performance IQ, ADHD: Attention Deficit Hyperactivity Disorder, ASD: Autism Spectrum Disorder, ODD: Oppositional Defiant Disorder*.* | | | | | | | | |

| *C)* | DCDQ total score | FSIQ | VIQ | PIQ | ADHD symptom Count | ASD trait symptom count | Anxiety symptom count | ODD symptom count |
| --- | --- | --- | --- | --- | --- | --- | --- | --- |
| DCDQ total score |  | 0.486^***^ | 0.430^***^ | 0.454^***^ | -0.453^***^ | -0.688^***^ | -0.270^***^ | -0.240^***^ |
| FSIQ |  |  | 0.920^***^ | 0.904^***^ | -0.262^***^ | -0.346^***^ | -0.148^*^ | -0.126 |
| VIQ |  |  |  | 0.671^***^ | -0.270^***^ | -0.369^***^ | -0.139^*^ | -0.144^*^ |
| PIQ |  |  |  |  | -0.204^**^ | -0.265^***^ | -0.125 | -0.072 |
| ADHD symptom Count |  |  |  |  |  | 0.594^***^ | 0.477^***^ | 0.520^***^ |
| ASD Trait symptom count |  |  |  |  |  |  | 0.432^***^ | 0.364^***^ |
| Anxiety symptom count |  |  |  |  |  |  |  | 0.420^***^ |
| ODD symptom count |  |  |  |  |  |  |  |  |
| Computed correlation used Pearson-method with pairwise-deletion, *p<.05, **p<.01, ***p<.001. DCDQ: Developmental Coordination Disorder Questionnaire, FSIQ: Full Scale IQ, VIQ: Verbal IQ, PIQ: Performance IQ, ADHD: Attention Deficit Hyperactivity Disorder, ASD: Autism Spectrum Disorder, ODD: Oppositional Defiant Disorder*.* | | | | | | | | |
